# Acute abdomen in patients with covid-19: an integrative review

**DOI:** 10.1590/0100-6991e-20233576-en

**Published:** 2023-08-14

**Authors:** MANUELA IZIDIO DE LIMA, OLIVAL CIRILO LUCENA DA FONSECA

**Affiliations:** 1 - Universidade de Pernambuco, Faculdade de Ciências Médicas - Recife - PE - Brasil; 2 - Universidade de Pernambuco, Hospital Universitário Oswaldo Cruz (HUOC) - Recife - PE - Brasil

**Keywords:** Acute Abdomen, COVID-19, SARS-CoV-2, Abdominal Pain, Abdome Agudo, COVID-19, SARS-CoV-2, Dor Abdominal

## Abstract

**Introduction:**

upon infection with SARS-CoV-2, patients presented with non-classical symptoms, such as gastrointestinal phenomena including loss of appetite, nausea, vomiting, diarrhea, and abdominal pain abdominal pain. These occurrences, typically, were found in severely affected patients with COVID-19. With this, the aim of this paper is to analyze the available knowledge on the development of acute abdomen in SARS-CoV-2 infected patients.

**Methodology:**

this is an Integrative Review in PubMed, Web of Science and VHL databases. The following descriptors were used: “Acute abdomen”, “COVID-19”, “Abdominal pain” and “SARS-CoV-2” with the Boolean operator “AND”, and articles relevant to the theme were selected. Initially, 331 articles were selected, all published between 2020 and 2023, in Portuguese and/or English. After analysis, 11 articles matched the proposed objective.

**Results:**

the relationship between tenderness in the right upper region or the presence of Murphy’s sign contributed in the association between abdominal pain and the more severe forms of COVID-19 in infected patients. The number of diagnoses for acute conditions such as cholecystitis, appendicitis, diverticulitis and pancreatitis decreased with the pandemic, but at the same time there was an increase in the duration of surgical procedures and in the length of hospital stays. These acute abdominal conditions were the result of delayed demand for hospital care, which also contributed to an increase in the conversion rate to open surgery and in the number of perforative conditions.

**Conclusion:**

the development of acute abdomen in SARS-CoV-2 infected patients was predictive of an unfavorable prognosis.

## RESUMO

**Introdução:**

a partir da infecção com o SARS-CoV-2, os pacientes apresentaram sintomas não clássicos, como fenômenos gastrointestinais que incluem perda de apetite, náuseas, vômitos, diarréia e dores abdominais. Essas ocorrências, normalmente, foram encontradas em pacientes gravemente afetados pelo COVID-19. Com isso, o objetivo deste trabalho é analisar o conhecimento disponível sobre o desenvolvimento do abdome agudo em pacientes infectados pelo SARS-CoV-2.

**Metodologia:**

trata-se de uma Revisão Integrativa na base de dados PubMed, Web of Science e BVS. Foi utilizado os descritores: “Acute abdomen”, “COVID-19”, “Abdominal pain” e “SARS-CoV-2” com o operador booleano “AND”, e selecionados artigos de relevância para o tema. Inicialmente, foram selecionados 331 artigos, todos publicados entre 2020 e 2023, em português e/ou inglês. Após análise, 11 artigos corresponderam ao objetivo proposto.

**Resultados:**

a relação entre a sensibilidade na região superior direita ou a presença do sinal de Murphy contribuiu na associação entre dor abdominal e as formas mais graves do COVID-19 em pacientes infectados. O número de diagnósticos para quadros agudos como colecistite, apendicite, diverticulite e pancreatite tiveram diminuição com a pandemia, mas ao mesmo tempo houve aumento na duração dos procedimentos cirúrgicos e nos períodos de internações. Esses quadros de abdome agudo foram resultados da procura tardia de assistência hospitalar que colaborou, inclusive no aumento da taxa de conversão para a cirurgia aberta e no número de quadros perfurativos.

**Conclusão:**

o desenvolvimento do abdome agudo em pacientes infectados pelo SARS-CoV-2 foi preditor de um prognóstico desfavorável.

## INTRODUCTION

The COVID-19 pandemic caused by the severe acute respiratory syndrome coronavirus 2, SARS-CoV-2, spread across the world within months of the initial outbreak in China in 2019, triggering a sudden and substantial increase in hospitalizations. The most common described symptoms of COVID-19 are fever, cough, myalgia, fatigue, and dyspnea. However, despite the respiratory system being the main target of SARS-CoV-2 and the most prevalent complication being the evolution to an Acute Respiratory Distress Syndrome (ARDS), patients infected with COVID-19 may present with non-classical manifestations, such as gastrointestinal symptoms, which include loss of appetite, nausea, emesis, diarrhea, and abdominal pain. These abdominal manifestations, in turn, are present in approximately 3% to 39% of patients with SARS-CoV-2^1^.

The involvement of the gastrointestinal tract (GIT) is deemed to be mediated by the expression of angiotensin-converting enzyme 2 receptors, ACE2, which are the main receptors of SARS-CoV-2. This was verified from GIT biopsies that demonstrated the presence of coronavirus 2 RNA. The hypothesis of GIT involvement is then due to direct viral injury and/or inflammatory immune response that can result in malabsorption, imbalance of intestinal secretions, dysfunction of the intestinal mucosa, and activation of the enteric nervous system^2^.

Patients who develop the severe form of COVID-19 are more predisposed to an acute abdomen, with manifestations of pancreatitis, appendicitis, cholecystitis, diverticulitis, intestinal obstruction and ischemia, hemoperitoneum, or abdominal compartment syndrome^3^. However, surgical societies, including the American College of Surgeons (ACS), have published guidelines regarding the screening of elective cases, recommending the postponement of elective surgeries. In addition, state and hospital authorities encouraged patients infected with COVID-19 who had a low-severity condition to avoid emergency rooms during the height of the pandemic, to prioritize the care of high-severity patients and avoid infection and the spread of SARS-CoV-2. As a result, patients with COVID-19 and acute problems who sought hospital care late had a worsening of the abdominal disease, making surgical treatment the best option^3^.

Therefore, the objective of this study is to investigate the existence of a probable association between the development of acute abdomen in patients with COVID-19 through an analysis of the currently available literature.

## METHODS

This is an integrative literature review based on the following steps: 1) Identification of the theme and elaboration of the research question; 2) Establishment of criteria for inclusion and exclusion of studies; 3) Definition of the information to be extracted from the selected studies; 4) Critical analysis of the included studies based on the levels of evidence; 5) Discussion of results; and 6) Presentation of the integrative review^4^ ^,^ ^5^.

We formulated the research according to the PICO strategy, which represents an acronym for Patient, Intervention, Comparison, and Outcomes, the guiding question. We then developed the following research question: What is the relationship between the development of an acute abdomen in patients with COVID-19?

We systematically carried out the bibliographic search in the databases PubMed, Web Of Science, and Virtual Health Library (VHL), with the latter only comprising scientific articles from Medline and Lilacs. We used the following terms, validated by Health Sciences Descriptors (DeCS): “acute abdomen”, “COVID-19”, “abdominal pain”, and “SARS-CoV-2”. The terms were interchanged by the Boolean operator “AND”, and we selected articles from 2020 to 2023.

The databases’ operation peculiarities result in different ways of searching for scientific articles. In PubMed and Web Of Science, the descriptors were used and the searches were expanded to all fields, with 80 and 38 articles found, respectively; in VHL, the same descriptors were used, restricting the search to articles’ titles, abstracts, and subjects, finding 202 articles from Medline and 11 from Lilacs. In the end, 331 we preselected articles.

**Figure 1:**
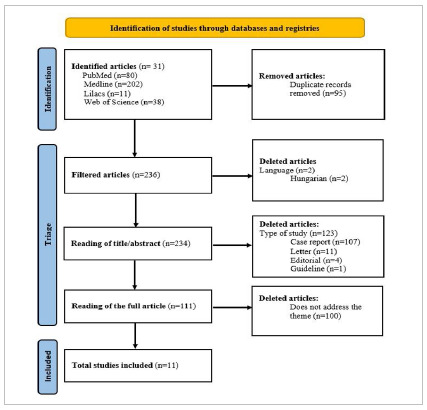
Screening of articles with the systematic review flowchart (PRISMA).

For the systematic selection of articles, we used the RAYYAN tool - Intelligent Systematic Review, considering the PRISMA Statement 2020 search strategy - Figure 1. As inclusion criteria, the articles should be complete, free to access, and in Portuguese or English. In addition, research should include studies describing the mechanism of GIT infection by SARS-CoV-2, the relationship between acute abdomen and SARS-CoV-2 infection, and the presentation, course, and prognosis of patients infected with SARS-CoV-2 who developed an acute abdomen. We also used the methodological rigor of the studies that demonstrate the quality of the manuscripts as an inclusion criterion, evaluating them through the levels of scientific evidence^4^. We excluded studies that did not answer the research question, that were incomplete, and the ones in languages other than Portuguese or English. Another exclusion criterion was studies that did not address the development of an acute abdomen in patients with COVID-19. At the end of the screening, we categorized the methodological quality of the included articles into levels I and II of scientific evidence, which means systematic reviews and meta-analyses, and retrospective and prospective studies, respectively. Table 1 describes the results.

**Table 1:** Classification of abdominal pain according to location and incidence rate in patients with COVID-19.

Abdominal pain	Location	Patients
Diffuse	-	43.7%
Localized	Epigastrium	42.7%
	Right hypochondrium	25.5%

[Table t2]

**Table 2 t2:** Relationship between abdominal pain and dyspnea in patients infected with SARS-CoV-2.

Abdominal pain location	Patients with dyspnea
Upper quadrant	63%
Lower quadrant	25.8%

[Table t3]

**Table 3 t3:** Summary of studies included in the integrative review.

Author	Journal/Year	Goal	Kind of study	Results	Evidence level
SEELIGER, B. et al.	Nature Public Health Emergency Collection/2020	To assess the underlying pathology and postoperative clinical course of emergency surgical patients infected with COVID-19.	Prospective study	Two groups were identified: group A, patients admitted to a hospital for an acute surgical condition with a concomitant diagnosis of COVID-19, and group B, patients with severe COVID-19 developing acute abdominal pathologies during their hospital stay. When compared with those in group B, patients in group A recovered overall better, with a lower mortality rate, lower rate of acute respiratory distress syndrome (ARDS), lower rates of preoperative invasive ventilation and postoperative invasive ventilation, and shorter duration of invasive ventilation.	II
Author	Journal/Year	Goal	Kind of study	Results	Evidence level
AZIZ, A. et al.	Cureus/2022	To assess clinical and laboratory features in adult patients with COVID-19 infection and acute pancreatitis (AP) and to determine if there is any relationship between AP and COVID-19 infection.	Systematic review	The development of AP from SARS-CoV-2 infection has been verified. The most common clinical presentation was abdominal pain.	I
BALAPHAS, A. et al.	Sci Rep./2022	To clarify patterns of abdominal pain related to SARS-CoV-2 infection during the pandemic and their relationships with serious clinical outcomes of COVID-19, modification of liver function tests (LFT), and other clinical features.	Retrospective study	The patients presented spontaneous abdominal pain and pain upon abdomen palpation. Spontaneous pain was most often located in the epigastric and right upper quadrant regions. Tenderness in the upper right region has been associated with severe COVID-19. Patients with a history of lower abdominal pain experienced dyspnea less frequently when compared with patients with a history of upper abdominal pain. Baseline elevation of transaminases was associated with a history of pain in the epigastrium and upper right region.	II
HABEEB, T. et al.	Int J Surg./2022	To assess the incidence, severity, and risk factors for recurrent appendicitis in patients treated without AI after successful AA drainage during the COVID-19 pandemic.	Prospective study	Recurrent appendicitis occurred in patients undergoing AA drainage during 1 year of follow-up. This is attributed to the fact that the COVID-19 pandemic induces vasculitis and thrombotic occlusion of arteries, including the appendicular artery. Additionally, the COVID-19 pandemic causes hyperplasia of the lymphoid tissue in the appendix wall, causing obstruction.	II
SERBAN, D. et al.	Exp The Med./ 2021	Investigated the effects of the COVID-19 pandemic on the clinical presentation and therapeutic management of the surgical acute abdomen.	Retrospective study	The main types of pathology in both groups included: Occlusions and peritonitis. SARS-CoV-2 infection can cause abdominal pain and should be considered in different diagnoses of acute abdomen.	II
KARIYAWASAM, J. et al.	Trans R Soc Trop Med Hyg./2021	Describe the gastrointestinal manifestations of COVID-19 and discuss the possible mechanisms and aspects related to its diagnosis and treatment.	Systematic review	Most gastrointestinal symptoms associated with COVID-19 include diarrhea, nausea, vomiting, and abdominal pain/discomfort. The patients had an acute abdomen with etiologies such as acute pancreatitis, acute appendicitis, intestinal obstruction, intestinal ischemia, hemoperitoneum, and abdominal compartment syndrome.	I
Author	Journal/Year	Goal	Kind of study	Results	Evidence level
HAYASHI, Y. et al.	J Gastroenterol./2021	Determine whether the presence of GI symptoms contributed to the severity of COVID-19 and identify GI symptoms characteristic of severe COVID-19.	Systematic review and meta-analysis	Abdominal pain is likely to be characteristic of severe COVID-19. Compared to other viral infectious diseases that primarily infect the respiratory system, patients with COVID-19 may have a slightly lower frequency of diarrheal symptoms with abdominal pain.	I
MCNABB-BALTAR, J. et al.	Am J Gastroenterol./2020	To assess the frequency and characteristics of hyperlipasemia in patients with COVID-19.	Retrospective study	Gastrointestinal symptoms were common among patients with hyperlipasemia, including nausea, general abdominal discomfort, and diarrhea. Among patients with hyperlipasemia, some required ICU admission and others died.	II
ALAKUS, Ü. et al.	Ulus Travma Acil Cerrahi Derg./2022	To examine the effect of the pandemic on the diagnosis and treatment of acute appendicitis.	Retrospective study	Patients who developed acute appendicitis during COVID-19 had the same clinical findings as patients who previously developed SARS-CoV-2 infection, but the frequency of presentation of such pathology was higher during the pandemic.	II
NICOLESCU, C. et al.	Surgery/2021	Analyze the difference between the surgical management of patients during the pandemic and non-pandemic periods, and determine the challenges and management of acute abdomen cases in the pandemic.	Retrospective study	The average time from admission to the surgical procedure was longer in the pandemic period, and there was an increase in operative time. The length of stay was longer in the pandemic period. Overall mortality more than doubled.	II
GEORGAKOPOULOU, V. et al.	Ann Saudi Med./2022	Review and analyze all reported cases of Acute Pancreatitis associated with COVID-19, reporting demographic data, clinical features, laboratory findings, and results.	Systematic review	Lipase and amylase were greater than three times the upper limit of normal (ULN), while the white blood cell count was elevated in most cases. The most frequent gastrointestinal, respiratory, and general symptoms were abdominal pain, dyspnea, and fever, respectively.	I

[Table t4]

**Table 4 t4:** Relationship between dysfunctions caused by SARS-CoV-2 infection, location of abdominal pain, and probability of dyspnea, duration of ICU, and death.

Dysfunction	Dyspnea, ICU, Death	Pain location
Hepatic	Higher probability	Right hypochondrium
Intestinal	Lower probability	Epigastrium
		Lower hemiabdomen

## RESULTS

From the analysis of the articles, the most common symptoms presented in patients positive for the SARS-CoV-2 virus were fever, dyspnea, and abdominal pain, with varied manifestations. The relationship between sensitivity in the upper right region or the presence of Murphy’s sign contributed to the association between abdominal pain and the most severe forms of COVID-19. By adjusting the patient’s need for abdominal surgery during hospitalization, the presentation of dyspnea, sex, and age group, these conditions became predictors with more chances of being associated with severe COVID-19^6^.

In the general population, length of stay and mortality of patients with acute abdomen manifestations were higher during the pandemic. The number of diagnoses for acute conditions such as cholecystitis, appendicitis, diverticulitis, pancreatitis, incarcerated hernias, intestinal occlusion, and perineal abscesses decreased with the pandemic, but the duration of the surgical procedure increased with the arrival of the pandemic. This is the result of the late search for hospital care, which contributed to an increase in the conversion rate to open surgeries and in the number of perforations. For acute appendicitis, for example, there were fewer cases of uncomplicated acute appendicitis and more cases of perforated and gangrenous ones^7^.

In addition, approximately one-third of patients had recurrent appendicitis that was related to abscess size, diabetes mellitus, and SARS-CoV-2 infection. Cases of severe diverticulitis were more frequent in patients with COVID-19. In pancreatitis, serum levels of amylase and lipase ranged from three times the upper limit of normal (ULN) to levels below three times the ULN in patients infected with SARS-CoV-2. Some patients with COVID-19 have had elevated white blood cell counts and C-reactive protein levels. In imaging studies, computed tomography and magnetic resonance imaging, abdominal findings were compatible with acute interstitial edematous pancreatitis in these patients. This acute abdomen condition, in addition to being related to the SARS-CoV-2 infection, was the result of the late search for hospital care, which also helped to increase the conversion rate to open surgery and the number of perforating conditions^6^ ^-^ ^12^.

Two situations were identified. A group with patients hospitalized for an acute abdominal condition in which a COVID-19 co-infection has been detected, called group A, and a group of patients hospitalized for a severe COVID-19 infection with a digestive complication requiring emergency surgery, called group B. When compared with group B, group A recovered better and had a lower rate of mortality, ARDS, and preoperative and postoperative invasive ventilation, with shorter duration^13^.

In group A, surgery was required within 24 hours of hospital admission due to incarcerated hernia, appendicitis, and pneumoperitoneum with peritonitis. Postoperatively, some patients required oxygen therapy and others required invasive ventilation due to ARDS. Overall, most patients recovered uneventfully, and some were discharged about six days after the operation. However, two patients had complications, one due to sepsis and radiological drainage of an intraperitoneal abscess, and the other died of septic shock on the night of surgery. The mortality rate in this group was one in seven patients^13^.

In group B, some patients were operated after 14 days of hospitalization. The pathologies presented were perforated duodenal ulcer, small intestine ischemia, sigmoid colon ischemia, and retroperitoneal and intraperitoneal hematoma. Some patients had preoperative invasive ventilation for more than seven days, and all surgical patients required postoperative invasive ventilation for ARDS. Complications were more severe and more frequent, including septic shock and renal failure. The mortality rate in this group was two out of six patients^13^.

## DISCUSSION

### Mechanism of infection of the gastrointestinal tract by SARS-CoV-2

Viral infection by SARS-CoV-2 to host cells occurs through binding to the angiotensin-converting enzyme 2 (ACE2) receptor on the cell surface, followed by activation of the spike (S) protein by transmembrane serine protease 2 (TMPRSS2). Virus entry into the cell is pre-activated by a target cell (TC) proprotein convertase called furin that reduces viral dependence on TC proteases for cell entry. Furin is found in the lungs, liver, pancreas, and GIT, and allows the virus to efficiently enter cells, avoiding immune surveillance and promoting transmission^8^ ^,^ ^14^.

After virus entry into the host cell, injury occurs by direct cell damage mediated by viral dysregulation of the renin-angiotensin-aldosterone system (RAAS). This happens because of down-regulation of ACE-2 related to viral entry, leading to decreased cleavage of angiotensin I and II. There is the beginning of an inflammatory response from the development of the cytokine storm, which leads to damage to endothelial cells and thrombo-inflammation, resulting in micro and macrovascular thromboses. In addition, the ACE2 receptor and the virus undergo endocytosis, leading to a reduction in ACE2 levels on the cell surface. This reduction interferes with amino acid homeostasis, with antimicrobial peptide expression, and with the ecology of the intestinal microbiome, which may increase inflammation^2^ ^,^ ^15^.

The liver and pancreas also express ACE2 receptors, even at higher levels than those expressed in the lungs. This makes these organs targets of SARS-CoV-2 and may be harmed by the direct cytotoxic effect of the virus through ACE2 receptors on cells or by the induced cytokine storm^6^.

### Presentation of acute abdomen in patients infected with SARS-CoV-2

The clinical manifestations of the acute abdomen most seen during the COVID-19 pandemic in the general population were obstructions, peritonitis, intra-abdominal or exteriorized hemorrhages at the level of the GIT, entero-mesenteric ischemia, hemoperitoneum, and abdominal compartment syndrome. All had a severe clinical course, sometimes even fatal, in the absence of surgical resolution due to the more advanced condition^2^ ^,^ ^9^.

Microthrombus is one of the characteristic pathophysiology presented in patients with COVID-19 and can result in ischemic changes secondary to thrombosis due to SARS-CoV-2 infection. This pattern of ischemia due to thrombosis can be seen in the intestinal region and is usually indicative of severe COVID-19^8^. The thrombotic etiology may be associated with direct viral invasion of the vascular endothelium or occlusion, resulting from the formation of microthrombi, which may cause ischemia of mesenteric vessels and infarctions of renal vessels. Non-thrombotic causes include acute pancreatitis, cholecystitis, diverticulitis, appendicitis, peritonitis, colonic distention, and colitis, and are related to the tropism of the virus to ACE receptors along the GIT^8^ ^,^ ^9^ ^,^ ^16^ ^,^ ^17^.

The expression of ACE2 receptors can lead to damage to pancreatic cells, hepatocytes, gallbladder mucosa, and proximal and distal enterocytes of the small intestine, with greater expression in the brush border of intestinal enterocytes and in absorptive enterocytes of the ileum and colon than in the lung. The direct cytotoxic action of SARS-CoV-2 or the indirect systemic inflammation mediated by the immune system may be the pathogenesis mechanisms of these lesions^2^ ^,^ ^11^ ^,^ ^12^ ^,^ ^15^.

### Presentation, evolution, and prognosis of patients infected with SARS-CoV-2 who developed an acute abdomen

In patients infected with SARS-CoV-2 who developed an acute abdomen and who underwent surgical interventions, this condition was a predictor of postoperative complications, such as disease progression and higher mortality rate. However, most patients who required surgical treatment upon hospital admission had favorable results^13^ ^,^ ^18^.

In patients infected with SARS-CoV-2 in a severe stage, there was a frequent report of pain, as per the Murphy sign, percussion and palpation, in the epigastric region, in the right hypochondrium, in the right inguinal region, and sensitivity in the left inguinal region. These are characteristic regions of liver, appendix, small intestine, and colon dysfunctions^6^.

SARS-CoV-2 infection induces a change in hepatic blood flow as a systemic response to the infection, with thrombi in the sinusoids and direct replication of the virus in the hepatic tissue. This may explain the elevation of liver function tests (LFT) observed during COVID-19. Dyspnea was more frequent in cases that presented with abdominal pain in the upper quadrant^6,19 21^.

In patients with COVID-19 during the pandemic, recurrent appendicitis was present in 30% of patients who underwent drainage of appendiceal abscess during one year. This is explained by SARS-CoV-2 infection inducing vasculitis, thrombotic occlusion of arteries, including the appendicular artery, and lymphoid tissue hyperplasia in the appendix wall, causing obstruction. In addition, patients diagnosed with appendicitis during the pandemic period had a more advanced radiological stage, which was attributed to the late search for hospital care^3^ ^,^ ^10^ ^,^ ^22^ ^-^ ^24^.

In patients with COVID-19 and acute pancreatitis (AP), the most common symptoms are abdominal pain, followed by dyspnea and fever. Treatment is typical for AP in non SARS-CoV-2 infected patients and includes intravenous fluids, analgesics, antiemetics, and antiviral treatment for COVID-19 infection. The elevation of serum amylase and lipase, despite being used as markers of pancreatic inflammation, may be related to other gastrointestinal pathologies, such as gastroparesis, gastritis, colitis, and cholecystitis, which are also recognized as part of the clinical picture of COVID-19^2^ ^,^ ^25^.

Diverticulitis is the inflammation of the inner wall of the large intestine, usually in the sigmoid colon region, and is characterized by the formation of protruding pockets called diverticula that, when inflamed, cause the disease. The usual symptoms include abdominal pain in the left groin, tenderness, and fever. During the pandemic, while severe cases and length of stay increased, mild episodes decreased^26^.

## CONCLUSION

The relationship between the manifestation of the acute abdomen in patients infected with SARS-CoV-2 was a predictor of an unfavorable prognosis. Cases with abdominal pain located in the right hypochondrium showed alterations in transaminase levels and increased risk of dyspnea, ICU admission, and death. The decrease in demand for hospital care due to isolation guidelines minimized the initial symptoms of acute conditions, resulting in a late search for medical help. Consequently, more severe cases of acute abdomen-related illnesses have become more frequent.
